# Acute kidney injury as a prognostic determinant in cardiogenic shock: a cohort study

**DOI:** 10.1007/s10157-025-02787-5

**Published:** 2025-11-12

**Authors:** Priyanka Boettger, Henriette Preusse-Sondermann, Jamschid Sedighi, Utku Bayram, Henning Lemm, Samuel Sossalla, Michael Buerke

**Affiliations:** 1 Department of Internal Medicine I, Cardiology, Critical Care Medicine, Justus-Liebig University, Giessen, Germany; 2 Department of Internal Medicine II, Cardiology, Angiology and Critical Care Medicine, St. Marien Hospital, Kampenstr. 51, 57072 Siegen, Germany; 3 Department of Internal Medicine II, Nephrology, Justus-Liebig University, Giessen, Germany

**Keywords:** Cardiogenic shock, Acute kidney injury, Renal replacement therapy, Risk stratification, Vasopressors, Mortality

## Abstract

**Background:**

Cardiogenic shock (CS) after myocardial infarction remains associated with high mortality. Acute kidney injury (AKI), a common complication, substantially impacts outcomes. We investigated the prognostic relevance of AKI and renal replacement therapy (RRT) in CS.

**Methods:**

In this retrospective study, 369 patients with infarct-related CS admitted to a tertiary center were analyzed. AKI was defined by KDIGO criteria. Clinical, laboratory, and hemodynamic data, including RRT use and in-hospital outcomes, were evaluated. Multivariable logistic regression identified independent predictors of AKI and RRT. Discriminatory power was assessed using AUC.

**Results:**

AKI occurred in 42.8% of patients (n = 143), with 60.1% developing AKI within 48 h and 35.0% classified as stage 3. AKI patients were older (70.5 vs. 67.2 years; p = 0.010), had more pre-existing CKD (100 vs. 83.3%; p = 0.002), and required longer ventilation (168 vs. 65.5 h; p < 0.001). Inflammatory, renal, and perfusion markers were significantly elevated from day 2 onward. RRT was initiated in 8.9% overall and 23.1% of AKI patients, with 60.6% mortality. Predictors of AKI included age (OR 2.40; 95% CI 1.10–5.12) and norepinephrine dose (OR 1.001 per µg/kg; p = 0.042; AUC = 0.71). Predictors of RRT were admission creatinine (OR 2.05 per mg/dL; p = 0.003) and absence of CPR (OR 0.22; p = 0.008; AUC = 0.75). Overall mortality was 57.7%, higher in women (66.4% vs. 53.4%; p = 0.021).

**Conclusions:**

AKI is common in infarct-related CS and linked to poor outcomes. Early identification of high-risk patients may enable timely renoprotective strategies.

## Introduction

Cardiogenic shock (CS) is a state of critical circulatory failure due to primary cardiac dysfunction, resulting in insufficient cardiac output to meet systemic metabolic demands and causing end-organ hypoperfusion and tissue hypoxia. Despite significant advances in early revascularization, pharmacologic support, and mechanical circulatory support (MCS), CS remains associated with high mortality, with 30-day rates often exceeding 40% and 1-year mortality approaching 50% [1–3]. CS most commonly results from acute myocardial infarction (AMI) but can also arise from decompensated heart failure, severe valvular disease, arrhythmias, or mechanical complications of infarction [4]. Contemporary definitions acknowledge CS as a dynamic spectrum, and the Society for Cardiovascular Angiography and Interventions (SCAI) classification provides a widely adopted framework for staging and clinical decision-making [5, 6].

Beyond the heart, extracardiac organ dysfunction is common and prognostically significant in CS. Among affected organs, the kidney is particularly vulnerable. Acute kidney injury (AKI) frequently complicates the course of CS, with reported incidences ranging from 13% to over 60%, depending on the population and AKI definition [7, 8]. The pathophysiology of AKI in CS is multifactorial: reduced cardiac output lowers renal perfusion pressure, while elevated central venous pressure contributes to renal venous congestion and impaired glomerular filtration [9, 10]. Concurrent neurohormonal activation—including stimulation of the renin-angiotensin-aldosterone system, sympathetic nervous system, and vasopressin pathways—further increases renal vascular resistance and promotes fluid retention, exacerbating renal dysfunction [11, 12].

The presence and severity of AKI are independently associated with poor outcomes, including higher short-term and long-term mortality, prolonged ICU stay, and increased risk of complications such as sepsis and bleeding [7, 8]. Stage 3 AKI and the need for renal replacement therapy (RRT) in particular portend markedly worse prognosis [13]. Despite this, data on AKI specifically in infarct-related CS remain limited.

In this retrospective observational study, we aimed to assess the incidence, timing, and clinical predictors of AKI in a cohort of patients with infarct-related CS treated at an academic hospital center. We also examined the association of AKI with in-hospital mortality and explored clinical characteristics and outcomes related to the use of RRT [14]. Particular attention was given to short-term outcomes, including need for mechanical ventilation, duration of intensive care, and in-hospital death, as well as the differential impact of AKI severity on prognosis.

## Methods

### Study design and setting

We conducted a retrospective, single-center cohort study at an academic tertiary care hospital in Germany. Medical records of patients admitted to the cardiac intensive care unit (ICU) between January 2010 and July 2015 were reviewed. The study protocol was approved by the institutional ethics committee (reference number FF55/2018) and complied with the principles of the Declaration of Helsinki.

### Patient population

Patients were eligible for inclusion if they were aged ≥ 18 years and admitted to the ICU with infarct-related cardiogenic shock. Cardiogenic shock was defined as systolic blood pressure < 90 mmHg for ≥ 30 min or the need for vasopressor therapy, in conjunction with signs of end-organ hypoperfusion (e.g., oliguria, elevated lactate, altered mental status) in the context of an acute myocardial infarction [13, 15].

Myocardial infarction was diagnosed based on elevated cardiac troponin levels above the 99th percentile upper reference limit and/or electrocardiographic evidence of ischemia (e.g., ST-elevation or new left bundle branch block), according to the universal definition of myocardial infarction [16]. Cardiopulmonary resuscitation (CPR) was recorded for all patients with cardiac arrest prior to or during hospitalization. Unless specified otherwise, CPR refers to all resuscitation events irrespective of location. When relevant for subgroup analyses, CPR was further classified as out-of-hospital or in-hospital.

### Exclusion criteria

The exclusion criteria I comprised end-stage renal disease (eGFR < 15 mL/min/1.73 m^2^) or maintenance dialysis, history of kidney transplantation, admission for non–infarct-related shock (e.g., septic, hypovolemic), death within 6 h of ICU admission, and incomplete renal function data.

### Definitions and clinical variables

AKI was defined in accordance with the Kidney Disease: Improving Global Outcomes (KDIGO) criteria [17, 18]: an increase in serum creatinine ≥ 0.3 mg/dL within 48 h, or ≥ 1.5 times the baseline within 7 days, or urine output < 0.5 mL/kg/h for ≥ 6 h. Baseline renal function was defined by the first available serum creatinine on admission. Estimated glomerular filtration rate (eGFR) was calculated using the CKD-EPI equation [19]. Urine output criteria were recorded only for a subset of patients and therefore not used for AKI staging in the present analysis. Left ventricular ejection fraction (LVEF) was measured by transthoracic echocardiography using the biplane Simpson’s method and categorized according to current ESC/AHA definitions as HFrEF (≤ 40%), HFmrEF (41–49%), and HFpEF (≥ 50%). In patients with previously reduced LVEF ≤ 40% showing recovery > 40%, the category HFimpEF was applied [20, 21].

Collected data included demographic variables, cardiovascular risk factors, comorbidities (e.g., chronic kidney disease, diabetes, hypertension), laboratory values (creatinine, CRP, lactate), hemodynamic parameters on ICU admission, use of vasopressors and inotropes, mechanical ventilation, and coronary angiography. Shock severity was assessed using the Sequential Organ Failure Assessment (SOFA) score on admission [22].

### Renal replacement therapy

Initiation of Renal replacement therapy (RRT) was based on clinical judgment in cases of volume overload, refractory hyperkalemia, metabolic acidosis, or uremia according to international guideline consensus [23]. Timing of initiation was recorded in hours after ICU admission. The primary modality used was continuous veno-venous hemodialysis (CVVHD); intermittent hemodialysis (IHD) was reserved for hemodynamically stable patients. RRT modality and indication were documented [24].

### Outcomes

The primary outcome was all-cause in-hospital mortality. Secondary outcomes included the incidence and timing of AKI, requirement for RRT, and ICU length of stay. signs of end-organ hypoperfusion (e.g., oliguria, altered mental status), and evidence of myocardial infarction [25, 26].

### Statistical analysis

After validation, data were exported from Microsoft Excel to SPSS Statistics (Version 24.0, IBM Corp., Armonk, NY, USA). Categorical variables were summarized as absolute and relative frequencies; continuous variables as means ± standard deviation or medians with interquartile range, depending on distribution. Group comparisons were performed using chi-square or Fisher’s exact test for categorical data, and t-tests or ANOVA for continuous variables, as appropriate. Pearson correlation coefficients were calculated to assess associations between continuous variables, interpreted as negligible (r < 0.1), weak (0.1–0.3), moderate (0.3–0.5), or strong (> 0.5). Multivariable logistic regression was used to identify independent predictors of binary outcomes, with results reported as odds ratios (ORs) and 95% confidence intervals (CIs). A two-sided p-value < 0.05 was considered statistically significant. Figures and tables were generated using SPSS, Excel, and Word (Office 365).

## Results

### Patient cohort and baseline characteristics

A total of 369 patients with infarct-related cardiogenic shock (CS) were included, of whom 66.9% (n = 247) were male. The mean age was 69.2 ± 12.2 years (range 26–93), with two missing values. Mean height was 171 ± 12.2 cm, weight 80.6 ± 13.8 kg, and BMI 27.3 ± 4.4 kg/m^2^; missing anthropometric data in 18 patients were imputed using national reference values. Patients were grouped by age: < 60 years (22.3%), 60–75 years (41.7%), and > 75 years (36.0%). At admission, 93.9% had systolic blood pressure < 90 mmHg. An eGFR < 30 mL/min/1.73m^2^ was seen in 25.3%, and serum lactate was elevated in 72.9% (mean 3.4 ± 3.2 mmol/L). Mean CRP was 7.2 ± 5.5 mg/dL, and leukocytosis was present in 60.2%. Preexisting CKD was documented in 84.8%, with 35.6% in KDIGO stage 3 and 1.8% in stage 5. Cardiovascular risk factors included hypertension (75.4%), diabetes (52.4%), hyperlipoproteinemia (55.8%), and obesity (63.8%). A family history of CAD was noted in 33.7%, smoking in 45.7%. Known CAD, prior myocardial infarction, and previous CABG were reported in 31.4%, 23.3%, and 15.3%, respectively. Chronic heart failure was diagnosed in 33.6%, across NYHA classes I–IV. In-hospital mortality was 57.7%, with an additional 1.1% dying within 30 days post-discharge. Mortality was significantly higher in women (66.4%) than men (53.4%), with an absolute sex difference of 13.0% (95% CI 2.0–24.0; p = 0.021). Baseline characteristics are detailed in Table 1. Peripheral artery disease and prior cerebrovascular events were present in 18.1 and 12.8% of patients, respectively, and moderate-to-severe valvular heart disease, predominantly aortic stenosis, was noted in 6.5%.

**Table 1:** Baseline characteristics of the study population (N = 369)

Variable	Value
Age, years (mean ± SD)	69.2 ± 12.2
Age groups	< 60 years: 22.3% (n = 82)
	60–75 years: 41.7% (n = 153)
	> 75 years: 36.0% (n = 132)
Sex	Male: 66.9% (n = 247)
	Female: 33.1% (n = 122)
Height, cm (mean ± SD)	171 ± 12.2
Weight, kg (mean ± SD)	80.6 ± 13.8
Body mass index, kg/m^2^ (mean ± SD)	27.3 ± 4.4
Smoking history (%)	45.7% (n = 163)
Known coronary artery disease (%)	31.4% (n = 101)
Prior myocardial infarction (%)	23.3% (n = 74)
Previous CABG surgery (%)	15.3% (n = 49)
Chronic heart failure (any NYHA class) (%)	33.6% (n = 124)
Hypertension (%)	75.4% (n = 260)
Diabetes mellitus (%)	52.4% (n = 182)
Hyperlipoproteinemia (%)	55.8% (n = 193)
Obesity (%)	63.8% (n = 217)
Family history of CAD (%)	33.7% (n = 116)
Chronic kidney disease (%)	84.8% (n = 313)
KDIGO stage 3 (%)	35.6%
KDIGO stage 5 (%)	1.8%
In-hospital mortality (%)	57.7% (n = 213)
30-day post-discharge mortality (%)	1.1% (n = 4)

### Clinical presentation and interventions in cardiogenic shock

**Fig. 1:**
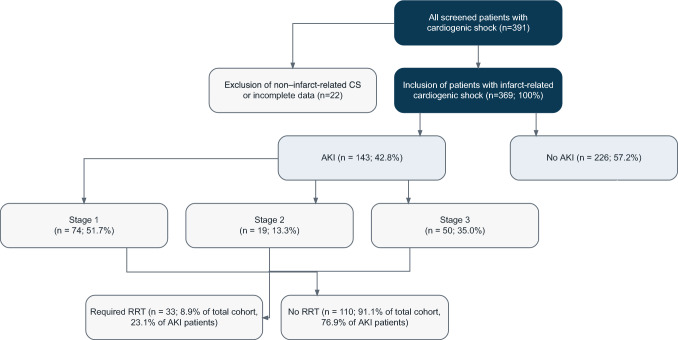
Patient selection and distribution of acute kidney injury (AKI) stages in infarct-related cardiogenic shock. A total of 391 patients presenting with cardiogenic shock were screened. After exclusion of 22 patients with non–infarct-related shock or incomplete data, 369 patients were included in the final cohort. AKI occurred in 143 patients (42.8%), distributed as KDIGO stage 1 in 74 (51.7%), stage 2 in 19 (13.3%), and stage 3 in 50 (35.0%). Renal replacement therapy (RRT) was required in 33 patients (8.9% of the total cohort; 23.1% of those with AKI), while 110 AKI patients (76.9%) did not receive RRT

At presentation, 93.9% of patients (n = 342) had a systolic blood pressure below 90 mmHg, and 79.7% (n = 283) were admitted with altered mental status. According to the Society for Cardiovascular Angiography and Interventions (SCAI) classification, 52.7% were classified as stage D, 34.2% as stage C, and 13.1% as stage E. Catecholamines were administered to 93.4% (n = 342), with norepinephrine doses increasing from 406 ± 936 μg/kg/day on day 1 to 1547 ± 4655 μg/kg/day on day 2. Mean arterial pressure (MAP) improved from 75.8 ± 20.8 mmHg on day 1 to 81.2 ± 13.2 mmHg on day 4 (p < 0.001, repeated-measures ANOVA) (Fig. 1).

Lactate at admission was elevated (mean 3.8 ± 3.6 mmol/L), indicating tissue hypoperfusion. Renal dysfunction was present at baseline in a substantial proportion, with 25.3% (n = 79) showing an estimated glomerular filtration rate (eGFR) < 30 mL/min/1.73 m^2^. Coronary angiography was performed in 74.9% (n = 274), and 48.8% underwent immediate reperfusion therapy (PCI or thrombolysis). Cardioversion or defibrillation was required in 48.2% (n = 173), and intra-aortic balloon pump (IABP) implantation in 24.2% (n = 64). Cardiopulmonary resuscitation (CPR) was performed in 248 patients (69.7%), including 164 out-of-hospital (44.4%) and 84 in-hospital events (22.8%). Mechanical ventilation was required in 71.8% (n = 265), with a mean duration of 106.2 ± 98.5 h. Cardiac index, measured in 13.0% (n = 48), was < 2.2 L/min/m^2^ in 35.4% (n = 17). Atrial fibrillation was present in 31.6%, and 20.2% exhibited ventricular tachycardia or fibrillation at admission. Inflammatory markers were elevated at baseline, with mean C-reactive protein (CRP) levels of 11.6 ± 9.8 mg/dL and leukocyte counts of 13.2 ± 5.5 × 10⁹/L. Median Sequential Organ Failure Assessment (SOFA) score on day 1 was 9 (interquartile range, 7–12), reflecting severe multiorgan dysfunction at presentation.

### Comparison of patients with and without acute kidney injury

Among 369 patients with infarct-related cardiogenic shock, 143 (42.8%) developed acute kidney injury (AKI) based on KDIGO criteria. AKI typically developed early during hospitalization, with more than half of events occurring within 48 h of admission and nearly four-fifths within the first 72 h, underscoring the acute nature of renal impairment in cardiogenic shock. Patients with AKI were significantly older (70.5 ± 9.8 vs. 67.2 ± 13.3 years; mean difference 3.3 years, 95% CI 0.8–5.9, p = 0.010) and more often had pre-existing chronic kidney disease (100 vs. 83.3%, p = 0.002) and hyperlipoproteinemia (62.4 vs. 49.4%, p = 0.021). Coronary angiography (83.2 vs. 73.7%, p = 0.038) and mechanical ventilation (79.0 vs. 68.1%, p = 0.026) were more frequent in the AKI group, with markedly longer ventilation times (168.0 ± 177.3 vs. 65.5 ± 110.0 h, p < 0.001). Inflammatory and perfusion markers were significantly elevated in AKI patients. On day 4, CRP (20.2 ± 11.8 vs. 14.8 ± 9.3 mg/dL, p = 0.002) and serum creatinine (2.6 ± 1.5 vs. 1.2 ± 0.5 mg/dL, p < 0.001) were higher. From day 2 onwards, lactate and norepinephrine requirements diverged (both p < 0.001), reflecting greater circulatory failure in the AKI group. Among AKI patients, 51.7% had stage 1, 13.3% stage 2, and 35.0% stage 3 AKI. Onset occurred within 24 h in 6.3%, between 24–48 h in 53.8%, 48–72 h in 28.0%, and 72–96 h in 11.9%. Clinical differences are summarized in Table 2.

**Table 2:** Comparison of clinical characteristics in patients with and without acute kidney injury (AKI)

Variable	No AKI (n = 191)	AKI (n = 143)	p-value
Age, years (mean ± SD)	67.2 ± 13.3	70.5 ± 9.8	0.01
Hyperlipoproteinemia (%)	49.4%	62.4%	0.021
Chronic kidney disease (%)	83.3%	100%	0.002
Mechanical ventilation (%)	68.1%	79.0%	0.026
Ventilation duration, h (mean ± SD)	65.5 ± 110.0	168.0 ± 177.3	< 0.001
Coronary angiography (%)	73.7%	83.2%	0.038
CRP on day 4, mg/dL (mean ± SD)	14.8 ± 9.3	20.2 ± 11.8	0.002
Creatinine on day 4, mg/dL (mean ± SD)	1.2 ± 0.5	2.6 ± 1.5	< 0.001
Lactate from day 2 onward	14.8 ± 9.3	20.2 ± 11.8	< 0.002
Norepinephrine dose (day 2 onward)	1.2 ± 0.5	2.6 ± 1.5	< 0.001

### Clinical characteristics according to AKI stage

**Fig. 2:**
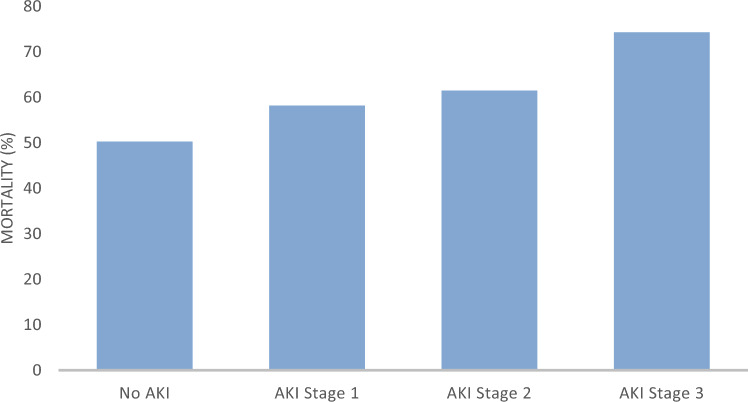
In-hospital mortality by AKI stage in infarct-related cardiogenic shock**-**Bar chart showing in-hospital mortality rates stratified by acute kidney injury (AKI) stage according to the KDIGO classification. A clear stepwise increase in mortality was observed with advancing AKI severity: from approximately 50% in patients without AKI to over 70% in those with AKI stage 3. This highlights the prognostic relevance of renal impairment in the setting of cardiogenic shock

Building upon the binary comparison between patients with and without AKI, Table 3 presents a stage-stratified analysis of clinical characteristics. Among patients with AKI, stage 1 was most frequent (51.7%), followed by stage 3 (35.0%) and stage 2 (13.3%). The proportion of patients aged > 75 years increased progressively with AKI severity, whereas BMI remained stable across stages. With increasing AKI severity, patients were older and more frequently had pre-existing chronic kidney disease and reduced baseline eGFR. A clear stepwise pattern of hemodynamic and metabolic deterioration was observed, reflected by rising lactate concentrations, lower arterial pH, and higher norepinephrine requirements across AKI stages (p < 0.001 for trend). The frequency and duration of mechanical ventilation likewise increased with worsening AKI. In contrast, exposure to contrast media during catheter intervention and the use of renin–angiotensin system inhibitors on admission were comparable across all stages (p > 0.05). Stage-specific in-hospital mortality rose from 41.9% in stage 1 to 72.0% in stage 3 (p for trend = 0.006; Fig. 2). Across AKI stages, patients exhibited rising inflammatory activity, increasing vasopressor requirements, and a progressive need for mechanical ventilation, underscoring the systemic nature of renal injury in cardiogenic shock. This clinical deterioration was mirrored by a stepwise increase in mortality with advancing AKI severity (Fig. 2).

**Table 3:** Comparison of clinical characteristics in patients across different AKI stages

Variable	No AKI (n = 226)	AKI 1 (n = 74)	AKI 2 (n = 19)	AKI 3 (n = 50)	*p*-value^*^ (trend)
Age (years), mean ± SD	65 ± 11	68 ± 10	70 ± 9	71 ± 9	0.04
Female sex, n (%)	56 (24.8)	21 (28.4)	6 (31.6)	15 (30.0)	0.72
Body-mass index (kg/m^2^)	27.3 ± 4.6	27.1 ± 4.5	26.8 ± 4.2	26.5 ± 4.1	0.59
Chronic kidney disease, n (%)	14 (6.2)	10 (13.5)	4 (21.1)	12 (24.0)	0.002
eGFR < 60 mL/min/1.73 m^2^, n (%)	38 (16.8)	20 (27.0)	7 (36.8)	21 (42.0)	0.001
Diabetes mellitus, n (%)	59 (26.1)	26 (35.1)	8 (42.1)	19 (38.0)	0.18
Hypertension, n (%)	139 (61.5)	51 (68.9)	14 (73.7)	37 (74.0)	0.22
Prior CAD, n (%)	81 (35.8)	33 (44.6)	10 (52.6)	24 (48.0)	0.19
Contrast exposure, n (%)	178 (78.8)	61 (82.4)	15 (78.9)	39 (78.0)	0.87
RAAS inhibitor use on admission, n (%)	112 (49.6)	33 (44.6)	7 (36.8)	20 (40.0)	0.36
Lactate (mmol/L), median [IQR]	3.2 [2.1–5.4]	4.1 [2.9–6.8]	5.0 [3.3–7.1]	6.2 [4.1–9.3]	< 0.001
Arterial pH, mean ± SD	7.34 ± 0.06	7.31 ± 0.07	7.29 ± 0.07	7.25 ± 0.08	< 0.001
Norepinephrine dose (µg/kg/min)	0.19 ± 0.09	0.23 ± 0.10	0.26 ± 0.11	0.30 ± 0.12	< 0.001
Mechanical ventilation, n (%)	114 (50.4)	47 (63.5)	14 (73.7)	41 (82.0)	< 0.001
IABP or Impella support, n (%)	89 (39.4)	34 (45.9)	9 (47.4)	25 (50.0)	0.31
Duration of mechanical ventilation (days), median [IQR]	3 [1–6]	5 [2–8]	6 [3–9]	7 [4–12]	< 0.001
In-hospital mortality, n (%)	45 (19.9)	24 (32.4)	9 (47.4)	31 (62.0)	< 0.001

^*^p-values indicate differences among AKI stages (No AKI, Stage 1, Stage 2, Stage 3) using one-way ANOVA or Kruskal–Wallis test for continuous variables and Chi-square test for categorical variables

### Ejection fraction and renal outcomes

**Fig. 3:**
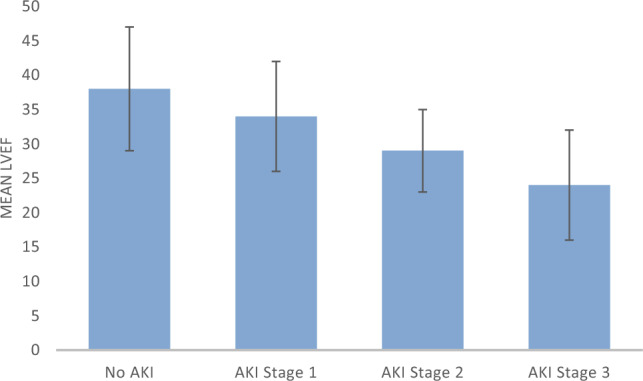
Left ventricular ejection fraction (LVEF) by AKI stage (KDIGO). Mean ± SD values are shown. LVEF declined stepwise with increasing AKI severity (No AKI: 38 ± 9%, AKI stage 1: 34 ± 8%, AKI stage 2: 30 ± 7%, AKI stage 3: 26 ± 7%; p < 0.001 for trend, ANOVA). Patients requiring renal replacement therapy (RRT) had the lowest mean LVEF (24 ± 7%). This progressive decline in systolic function across AKI stages illustrates the close hemodynamic–renal coupling characteristic of infarct-related cardiogenic shock

Left ventricular ejection fraction (LVEF) declined stepwise with increasing AKI severity (No AKI: 38 ± 9%, AKI 1: 34 ± 8%, AKI 2: 30 ± 7%, AKI 3: 26 ± 7%; p < 0.001 for trend, ANOVA; Fig. 3). Patients with AKI had lower LVEF than those without AKI (33 ± 8% vs. 38 ± 9%; p = 0.004), and lower LVEF remained independently associated with AKI (adjusted OR 1.25, 95% CI 1.10–1.42 per 5% decrement; p = 0.002). A severely reduced LVEF (< 30%) occurred in 44% of AKI versus 17% of non-AKI patients (p = 0.001). LVEF correlated inversely with peak serum creatinine (ρ = −0.42; p < 0.001), and patients requiring renal replacement therapy had the lowest mean LVEF (24 ± 7% vs. 34 ± 8%; p = 0.009). In-hospital mortality increased in parallel with both declining LVEF and rising AKI stage (34% in No AKI, 48% in AKI 1, 63% in AKI 2, and 74% in AKI 3; p = 0.006 for trend). This stepwise deterioration of systolic function and renal outcomes underscores the tight hemodynamic–renal coupling characteristic of infarct-related cardiogenic shock.

### Predictors of AKI and renal replacement therapy (RRT)

In multivariable logistic regression, advanced age (OR 2.40 per category; 95% CI 1.10–5.12; p = 0.025) and norepinephrine dose on day 1 (OR 1.001 per μg/kg; 95% CI 1.000–1.002; p = 0.042) were independently associated with AKI development. The model demonstrated moderate discrimination (AUC = 0.71, 95% CI 0.66–0.76).

To further delineate determinants of renal replacement therapy, patients who required RRT (n = 33) were compared with those managed conservatively (Table 4). Individuals needing RRT were older (71.5 ± 9.2 vs. 66.5 ± 11.5 years) and more frequently presented with hyperlipoproteinemia (84.4 vs. 52.9%, p = 0.001), a family history of coronary artery disease (50.0 vs. 32.1%, p = 0.041), peripheral artery disease (32.3 vs. 15.8%, p = 0.022), and pre-existing chronic kidney disease (100 vs. 83.3%, p = 0.002). Mechanical ventilation lasted significantly longer in the RRT group (172.9 ± 143.0 vs. 99.3 ± 150.9 h, p = 0.033). RRT patients exhibited persistently higher norepinephrine requirements, particularly on day 3 (1920.5 ± 1810.1 vs. 1072.8 ± 1530.6 μg/kg/day, p = 0.014) and day 4 (1602.2 ± 1676.9 vs. 707.9 ± 912.2 μg/kg/day, p = 0.018), reflecting sustained circulatory instability. Dobutamine use did not differ significantly. In-hospital mortality among RRT recipients reached 60.6%, underscoring the high-risk phenotype of severe AKI in cardiogenic shock. In logistic regression, admission creatinine (OR 2.05 per mg/dL; 95% CI 1.29–3.24; p = 0.003) and absence of specifically pre-hospital CPR (OR 0.22; 95% CI 0.07–0.67; p = 0.008) emerged as independent predictors of RRT need. The RRT prediction model achieved good discrimination (AUC = 0.75, 95% CI 0.68–0.81). Although in-hospital mortality was higher among RRT patients (60.6% vs. 57.4%), this difference was not statistically significant (p = 0.73). While RRT itself did not independently predict mortality, its initiation reflects severe multiorgan dysfunction and hemodynamic instability, serving as a surrogate marker of poor prognosis in cardiogenic shock.

**Table 4:** Baseline characteristics and clinical data of patients with and without renal replacement therapy (RRT)

Parameter	RRT (n = 33)	No RRT (n = 336)	p-value
Gender			0.259
Female, % (n)	24.2% (8)	33.9% (114)	
Male, % (n)	75.8% (25)	66.1% (222)	
Age groups			0.149
< 60 years, % (n)	9.1% (3)	23.7% (79)	
60–75 years, % (n)	51.5% (17)	40.7% (136)	
> 75 years, % (n)	39.4% (13)	35.6% (119)	
Mean age, years (± SD)	71.51 (± 9.21)	68.93 (± 12.46)	
Mortality			0.725
Survivors, % (n)	39.4% (13)	42.6% (143)	
Non-survivors, % (n)	60.6% (20)	57.4% (193)	
Cardiovascular risk factors			
Smoking, % (n)	51.5% (17)	45.2% (146)	0.488
Hypertension, % (n)	81.3% (26)	74.8% (234)	0.417
Diabetes mellitus, % (n)	56.3% (18)	52.1% (164)	0.651
Hyperlipoproteinemia, % (n)	84.4% (27)	52.9% (166)	**0.001**
Family history of CAD, % (n)	50.0% (16)	32.1% (100)	**0.041**
Obesity, % (n)	71.0% (22)	63.1% (195)	0.385
Pre-existing conditions			
Previous myocardial infarction, % (n)	33.3% (11)	22.1% (63)	0.148
Previous CABG surgery, % (n)	21.2% (7)	14.6% (42)	0.316
Known coronary artery disease, % (n)	27.3% (9)	31.8% (92)	0.593
Previous shock, % (n)	9.1% (3)	3.5% (10)	0.124
Previous ICD/CRT implantation, % (n)	6.3% (2)	4.9% (14)	0.736
Peripheral arterial disease, % (n)	32.3% (10)	15.8% (45)	**0.022**
Liver disease, % (n)	6.3% (2)	3.9% (11)	0.524
Pulmonary disease, % (n)	12.5% (4)	20.3% (58)	0.292
Pulmonary arterial hypertension, % (n)	3.1% (1)	3.2% (9)	0.984
Pre-existing chronic heart failure (NYHA)			0.925
NYHA class 1, % (n)	15.4% (2)	14.4% (16)	
NYHA class 2, % (n)	30.8% (4)	29.7% (33)	
NYHA class 3, % (n)	38.5% (5)	32.4% (36)	
NYHA class 4, % (n)	15.4% (2)	23.4% (26)	
Mean pre-existing EF, % (± SD)	50.43% (± 17.11)	49.23% (± 17.59)	0.810
Current mean EF, % (± SD)	42.74% (± 15.41)	41.95% (± 17.62)	0.804
Pre-existing chronic kidney disease (CKD)			**0.002**
Total CKD, % (n)	100% (33)	83.3% (280)	
CKD stage 1, % (n)	12.1% (4)	25.0% (70)	
CKD stage 2, % (n)	15.2% (5)	26.1% (73)	
CKD stage 3, % (n)	54.5% (18)	37.5% (105)	
CKD stage 4, % (n)	6.1% (2)	9.6% (27)	
CKD stage 5, % (n)	12.1% (4)	1.8% (5)	
Diagnostics and therapy			
Catecholamine administration, % (n)	93.9% (31)	92.6% (311)	0.904
CPR during hospitalization, % (n)	48.5% (16)	69.0% (232)	**0.011**
Coronary angiography, % (n)	81.8% (27)	74.2% (247)	0.334
Cardioversion/defibrillation, % (n)	48.5% (16)	48.2% (157)	0.972
Intra-aortic balloon pump (IABP), % (n)	16.0% (4)	25.0% (60)	0.317
Mechanical ventilation, % (n)	78.8% (26)	71.1% (239)	0.351
Duration of ventilation (h, ± SD)	172.94 (± 143.03)	99.27 (± 150.88)	**0.033**
Dialysis duration (mean h, ± SD)	87.09 (± 103.23)	–	–

*CAD* coronary artery disease, *CABG* coronary artery bypass graft, *CPR* cardiopulmonary resuscitation, *ICD* implantable cardioverter-defibrillator, *CRT* cardiac resynchronization therapy, *NYHA* new York Heart Association, *EF* ejection fraction, *CKD* chronic kidney disease, *IABP* intra-aortic balloon pump

### Multivariate analysis

**Fig. 4:**
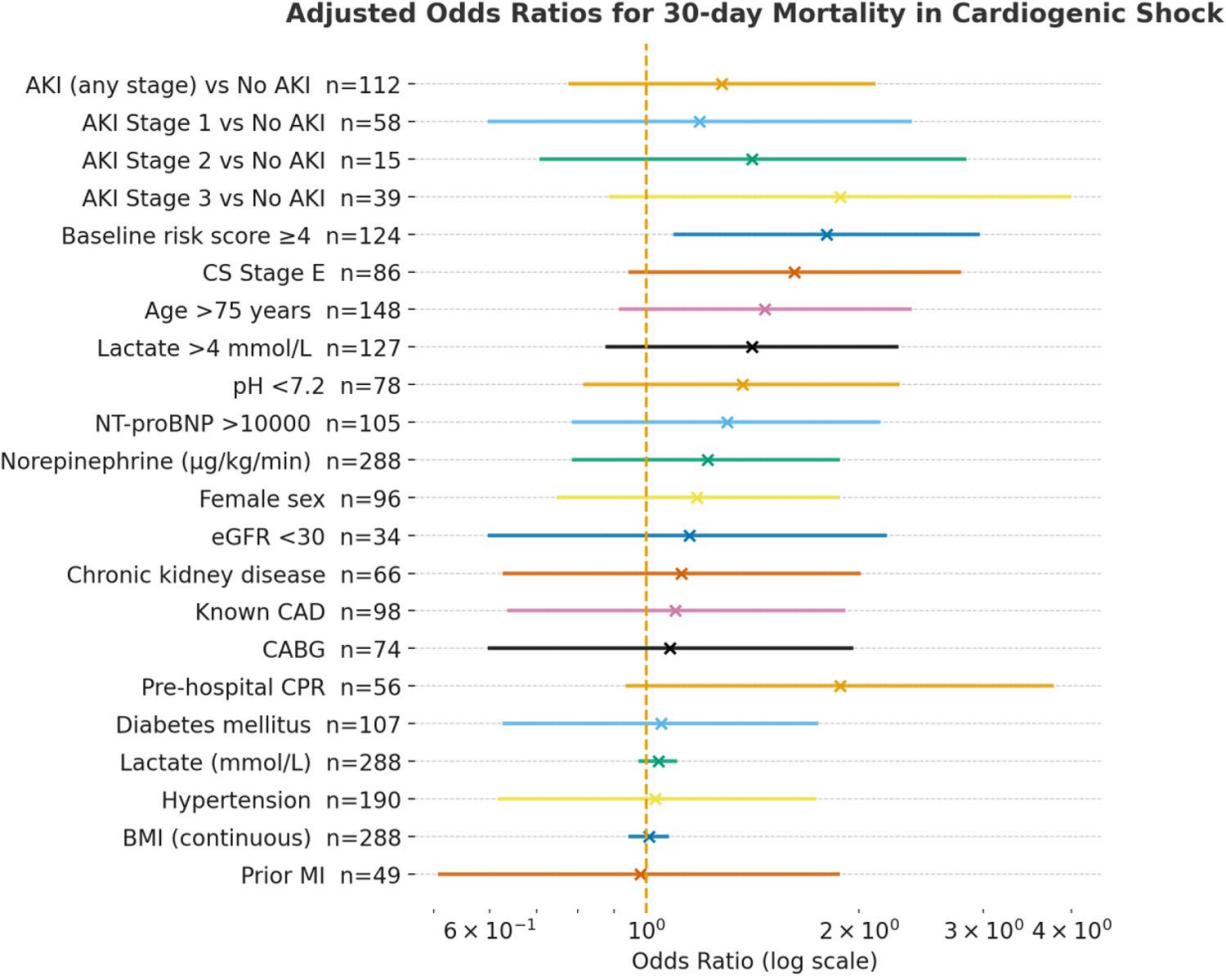
Forest plot displaying adjusted odds ratios (OR) for 30-day mortality in patients with infarct-related cardiogenic shock. Each marker denotes an independent variable from the multivariable logistic regression model; horizontal lines indicate 95% confidence intervals on a logarithmic scale. The vertical dashed line (OR = 1.0) represents the null effect. Numbers to the right of each variable indicate the number of complete cases included in the analysis. Variables encompass demographic and clinical characteristics, hemodynamic parameters, and renal function markers. Both “AKI (any stage) vs No AKI” and stage-stratified comparisons (“AKI 1–3 vs No AKI”) are displayed, illustrating a non-significant stage-dependent gradient. “Lactate (continuous)” indicates the risk increase per 1 mmol/L increment, whereas “Lactate > 4 mmol/L” reflects a dichotomous threshold analysis

In a separate multivariate logistic regression model with 30-day mortality as the dependent outcome, no variable retained statistical significance after full adjustment. (Fig. 4). However, pre-hospital CPR showed a strong trend toward increased mortality risk (OR 1.88, 95% CI 0.94–3.75; *p* = 0.07), whereas in-hospital CPR did not reach statistical significance (OR 1.21, 95% CI 0.63–2.34; *p* = 0.56). Similarly, age > 75 years, arterial pH < 7.2, and SCAI stage E were associated with numerically higher odds of death but did not reach significance. Notably, although both acute kidney injury (AKI) and elevated lactate were strong predictors in univariate analysis, they did not remain independently predictive after adjustment—potentially reflecting shared pathophysiological pathways and multicollinearity in critically ill patients with advanced shock. A full summary of the mortality model is presented in Fig. 4.

## Discussion

In this large, prospective, single-center cohort of 369 patients with infarct-related cardiogenic shock (CS), acute kidney injury (AKI) developed in 42.8% of cases and was clearly associated with significantly worse clinical outcomes, increased demand for organ support, and a markedly elevated in-hospital mortality rate of 74.1% in KDIGO stage 3. Our data reveal a stepwise rise in mortality with increasing AKI severity, from 50.2% without AKI to 74.1% in those with stage 3 AKI, affirming a graded risk relationship. It is a common and prognostically relevant complication in CS, reflecting the extent of hemodynamic compromise and systemic hypoperfusion [2]. Renal failure, most commonly manifesting as AKI, is a frequent and prognostically significant complication in CS [7, 11, 27]. The pathophysiology is multifactorial, involving reduced renal perfusion due to low cardiac output, increased venous congestion, neurohormonal activation, and exposure to nephrotoxic agents and mechanical circulatory support (MCS) devices [9]. Previous multicenter cohort studies have reported AKI incidences of up to 60% in heart failure–related CS and approximately 20% requiring renal replacement therapy (RRT) during hospitalization [7, 8, 28, 29]. Our observed incidence aligns with these findings and closely mirrors the CardShock registry, which also reported high rates of renal impairment in CS cohorts [30]. However, our study offers additional granularity by applying KDIGO stage-specific classification in a homogenous infarct-related CS cohort. Few studies to date have examined the dose-response relationship between AKI severity and mortality using validated staging in a non-postcardiotomy setting. By demonstrating a consistent rise in mortality across KDIGO stages and identifying predictors for AKI progression and RRT initiation, our study offers valuable clinical stratification tools. This analysis provides several novel contributions to the understanding of renal dysfunction in infarct-related cardiogenic shock. In contrast to prior multicenter cohorts that included heterogeneous etiologies of cardiogenic shock, the present study focuses exclusively on infarct-related cases, allowing for a pathophysiologically coherent interpretation of renal injury. Through systematic application of KDIGO staging, a distinct, stage-dependent gradient in mortality, organ dysfunction, and inflammatory activation was demonstrated, thereby extending previous reports that used binary AKI definitions. The integration of hemodynamic, metabolic, and inflammatory variables offers a mechanistic perspective linking renal deterioration to circulatory failure and systemic inflammation—an approach that deepens current insight into the cardiorenal axis. This mechanistic understanding is further supported by our clinical data, which delineate distinct predictors and patient profiles associated with renal dysfunction. Furthermore, identification of independent predictors for both AKI and RRT, including age, norepinephrine dose, and baseline creatinine, adds prognostic granularity within this infarct-specific cohort. Taken together, these findings establish KDIGO-based staging as a clinically applicable and prognostically powerful framework for early risk stratification and renal management in cardiogenic shock.

We identified advanced age and high vasopressor requirements as independent predictors of AKI, consistent with previous data from IABP-SHOCK trial, IABP-SHOCK II [31, 32] and CardShock [30]. Notably, our subgroup analyses further suggest that AKI was more common in patients with higher BMI, diabetes mellitus, hyperlipoproteinemia, and pre-existing chronic kidney disease. The extremely high rate of baseline CKD (84.8%) and the presence of KDIGO stage 3 in over one-third of patients underscore the interplay between chronic renal impairment and AKI development in CS [10, 28]. Nonetheless, pre-existing conditions such as hypertension and diabetes mellitus alone were not independently associated with higher mortality, highlighting the dominant role of acute hemodynamic stress and renal deterioration. Beyond creatinine-based AKI staging, proteinuria represents an early marker of renal injury and systemic congestion in cardiogenic shock [1, 33]. Prior studies have linked proteinuria to increased AKI risk and short-term mortality in acute heart failure and infarction-related shock [34, 35]. Although proteinuria was not systematically available in our dataset, its established prognostic role underscores the importance of integrating urinary biomarkers in future analyses.

The need for RRT was observed in nearly one-quarter of patients with AKI, often prompted by metabolic derangements and fluid overload. RRT recipients were typically older and more comorbid, with higher rates of hyperlipoproteinemia, family history of coronary artery disease, peripheral arterial disease, and prolonged ventilation times. The observed prolongation of mechanical ventilation in this subgroup likely reflects not only extended intensive care requirements but also a higher overall severity of illness and the occurrence of secondary complications, particularly ventilator-associated pneumonia and sepsis, both of which may amplify systemic inflammation and multiorgan dysfunction in cardiogenic shock [33, 36, 37].

Despite aggressive organ support, their prognosis remained poor, reinforcing the complex clinical challenge of managing severe AKI in CS [38, 39]. Admission creatinine levels and absence of cardiopulmonary resuscitation emerged as independent predictors of RRT, emphasizing the relevance of baseline renal reserve and initial cardiac arrest status for early nephrology decision-making [40].

From a therapeutic standpoint, continuous renal replacement therapy remains the modality of choice due to its hemodynamic tolerability and ability to gradually manage volume status and solutes [5]. However, our data suggest that the mere initiation of RRT—regardless of modality—is a strong prognostic signal for poor outcome. As such, emphasis should be placed on early recognition of AKI trajectory, personalized thresholds for RRT initiation, and targeted prevention of secondary renal insults.

Early renal protection in cardiogenic shock centers on rapid hemodynamic stabilization, meticulous fluid balance management, and avoidance of nephrotoxic agents. Current guidelines recommend maintaining a mean arterial pressure ≥ 65 mmHg to preserve renal perfusion, with norepinephrine as the preferred first-line vasopressor [13, 41] Individualized volume management is crucial, as positive fluid balance and venous congestion are independently linked to higher mortality [1, 42]. Mechanical circulatory support, such as Impella, may enhance renal perfusion by improving cardiac output and reducing renal resistance [27]. Continuous renal replacement therapy remains preferable in hemodynamically unstable patients [41]. Integrating these measures into routine shock management may mitigate renal injury and improve outcomes [43].

Our study further contributes to the growing understanding of the cardiorenal axis in CS. The observed mortality gradient across KDIGO stages likely reflects cumulative renal injury from ischemia-reperfusion, neurohormonal maladaptation, venous congestion, and systemic inflammation [44, 45]. These findings position AKI as a key driver, not just a byproduct, of multiorgan failure and death. [46]**.** Importantly, our cohort comprises real-world patients uniformly diagnosed with infarct-related CS, without restriction to revascularized or mechanically supported cases. This broadens the applicability of our results, especially in secondary and tertiary care centers managing a high proportion of elderly, comorbid patients. Moreover, we provide one of the first comprehensive reports on AKI staging in infarct-related CS, supporting the prognostic value of KDIGO criteria in this critical population.

In conclusion, our study confirms and expands upon prior evidence linking AKI with adverse outcomes in CS [47]. We underscore AKI as a frequent, stage-dependent, and modifiable determinant of mortality. Clinical guidelines should emphasize the use of staging-based risk stratification**,** early renal consultation, and continuous renal function surveillance and early treatment in CS. Future research is needed to refine AKI phenotyping, validate renal biomarkers in this population, and define RRT thresholds tailored to hemodynamic and renal status [48]. Our findings advocate for integrative management strategies addressing both cardiac and renal dysfunction in CS to improve patient outcomes.

### Limitations

Several limitations should be acknowledged. First, this was a single-center retrospective analysis, limiting generalizability. Second, data on fluid balance and diuretic use were not uniformly available, which are important for AKI characterization. Third, the relatively small size of the RRT subgroup limits statistical power for mortality comparisons. Lastly, although we used logistic regression, residual confounding cannot be excluded. Urine output data were not systematically recorded in the source registry, limiting AKI classification to the creatinine-based KDIGO definition. Proteinuria measurements were not systematically available in the registry, which may have limited a more granular assessment of renal dysfunction and prognosis.

## Conclusions

Our study confirms that AKI is a frequent, stage-dependent, and powerful determinant of outcome in infarct-related cardiogenic shock. By applying KDIGO criteria in a real-world ICU cohort, we demonstrate a clear, incremental association between AKI severity and in-hospital mortality. Independent predictors such as older age, vasopressor need, elevated admission creatinine, and absence of in- and out-of-hospital CPR reflect underlying hemodynamic instability and systemic compromise. Patients requiring renal replacement therapy had substantially worse outcomes, highlighting the critical role of early identification and renoprotective strategies. These findings reinforce prior registry data [25, 30, 49] and emphasize the need to incorporate renal staging into prognostic models and clinical decision-making in cardiogenic shock.

## Data Availability

The datasets generated and/or analyzed during the current study are available from the corresponding author upon reasonable request.

## References

[1] Lüsebrink E, et al. Cardiogenic shock. Lancet. 2024;404(10466):2006–20. 39550175 10.1016/S0140-6736(24)01818-X

[2] Samsky MD, et al. Cardiogenic shock after acute myocardial infarction: a review. JAMA. 2021;326(18):1840–50. 34751704 10.1001/jama.2021.18323 PMC9661446

[3] Sinha SS, et al. 2025 Concise clinical guidance: an ACC expert consensus statement on the evaluation and management of cardiogenic shock: a report of the american college of cardiology solution set oversight committee. J Am Coll Cardiol. 2025;85(16):1618–41. 40100174 10.1016/j.jacc.2025.02.018

[4] Murphy A, Goldberg S. Mechanical complications of myocardial infarction. Am J Med. 2022;135(12):1401–9. 36075485 10.1016/j.amjmed.2022.08.017

[5] Blumer V, et al. Cardiogenic shock in older adults: a focus on age-associated risks and approach to management: a scientific statement from the American Heart Association. Circulation. 2024;149(14):e1051–65. 38406869 10.1161/CIR.0000000000001214 PMC11067718

[6] Henry TD, et al. Invasive management of acute myocardial infarction complicated by cardiogenic shock: a scientific statement from the American Heart Association. Circulation. 2021;143(15):e815–29. 33657830 10.1161/CIR.0000000000000959

[7] Sundermeyer J, et al. Kidney injury in patients with heart failure-related cardiogenic shock: results from an international, multicentre cohort study. Eur J Heart Fail. 2025. 10.1002/ejhf.3701 PMC12765233 40436616

[8] Abadeer AI, et al. Importance of stratifying acute kidney injury in cardiogenic shock resuscitated with mechanical circulatory support therapy. J Thorac Cardiovasc Surg. 2017;154(3):856-864.e4. 28554672 10.1016/j.jtcvs.2017.04.042

[9] Tang WHW, et al. Evaluation and management of kidney dysfunction in advanced heart failure: a scientific statement from the American Heart Association. Circulation. 2024;150(16):e280–95. 39253806 10.1161/CIR.0000000000001273

[10] McCallum W, Sarnak MJ. Cardiorenal syndrome in the hospital. Clin J Am Soc Nephrol. 2023;18(7):933–45. 36787124 10.2215/CJN.0000000000000064 PMC10356127

[11] Rangaswami J, et al. Cardiorenal syndrome: classification, pathophysiology, diagnosis, and treatment strategies: a scientific statement from the American Heart Association. Circulation. 2019;139(16):e840–78. 30852913 10.1161/CIR.0000000000000664

[12] Bruno RR, et al. Pharmacological treatment of cardiogenic shock–a state of the art review. Pharmacol Ther. 2022;240:108230. 35697151 10.1016/j.pharmthera.2022.108230

[13] van Diepen S, et al. Contemporary management of cardiogenic shock: a scientific statement from the American Heart Association. Circulation. 2017;136(16):e232–68. 28923988 10.1161/CIR.0000000000000525

[14] Claure-Del Granado R, Clark WR. Continuous renal replacement therapy principles. Semin Dial. 2021;34(6):398–405. 33819361 10.1111/sdi.12967

[15] Hochman JS, et al. Early revascularization in acute myocardial infarction complicated by cardiogenic shock. SHOCK investigators. should we emergently revascularize occluded coronaries for cardiogenic shock. N Engl J Med. 1999;341(9):625–34. 10460813 10.1056/NEJM199908263410901

[16] Thygesen K, et al. Third universal definition of myocardial infarction. Circulation. 2012;126(16):2020–35. 22923432 10.1161/CIR.0b013e31826e1058

[17] Kellum JA, Lameire N. Diagnosis, evaluation, and management of acute kidney injury: a KDIGO summary (part 1). Crit Care. 2013;17(1):204. 23394211 10.1186/cc11454 PMC4057151

[18] Khwaja A. Kdigo clinical practice guidelines for acute kidney injury. Nephron Clin Pract. 2012;120(4):c179–84. 22890468 10.1159/000339789

[19] Levey AS, et al. A new equation to estimate glomerular filtration rate. Ann Intern Med. 2009;150(9):604–12. 19414839 10.7326/0003-4819-150-9-200905050-00006 PMC2763564

[20] McDonagh TA, et al. 2023 Focused update of the 2021 ESC guidelines for the diagnosis and treatment of acute and chronic heart failure. Eur Heart J. 2023;44(37):3627–39. 37622666 10.1093/eurheartj/ehad195

[21] Maddox TM, et al. 2024 ACC expert consensus decision pathway for treatment of heart failure with reduced ejection fraction. JACC. 2024;83(15):1444–88. 38466244 10.1016/j.jacc.2023.12.024

[22] Vincent JL, et al. The SOFA (sepsis-related organ failure assessment) score to describe organ dysfunction/failure. on behalf of the working group on sepsis-related problems of the European Society of Intensive Care Medicine. Intensive Care Med. 1996;22(7):707–10. 8844239 10.1007/BF01709751

[23] Jourdain M, et al. Renal replacement therapy in an intensive care unit: guidelines from the SRLF-GFRUP consensus conference. Ann Intensive Care. 2025;15(1):100. 40668437 10.1186/s13613-025-01517-0 PMC12267776

[24] Barbar SD, Wald R, Quenot J-P. Acute kidney injury: when and how to start renal replacement therapy. Intensive Care Med. 2025;51(6):1172–5. 40387887 10.1007/s00134-025-07933-x

[25] Vahdatpour C, Collins D, Goldberg S. Cardiogenic shock. J Am Heart Assoc. 2019;8(8):e011991. 30947630 10.1161/JAHA.119.011991 PMC6507212

[26] Sinha SS, et al. 2025 Concise clinical guidance: an ACC expert consensus statement on the evaluation and management of cardiogenic shock. JACC. 2025;85(16):1618–41. 40100174 10.1016/j.jacc.2025.02.018

[27] Patsalis N, et al. Early risk predictors of acute kidney injury and short-term survival during Impella support in cardiogenic shock. Sci Rep. 2024;14(1):17484. 39080441 10.1038/s41598-024-68376-w PMC11289486

[28] Kataria R, et al. Worsening renal function is common and is associated with higher mortality rates in cardiogenic shock: a cardiogenic shock working group report. J Card Fail. 2025. 10.1016/j.cardfail.2025.03.012 40180238

[29] Adegbala O, et al. Characteristics and outcomes of patients with cardiogenic shock utilizing hemodialysis for acute kidney injury. Am J Cardiol. 2019;123(11):1816–21. 30967283 10.1016/j.amjcard.2019.02.038

[30] Tarvasmäki T, et al. Current real-life use of vasopressors and inotropes in cardiogenic shock–adrenaline use is associated with excess organ injury and mortality. Crit Care. 2016;20(1):208. 27374027 10.1186/s13054-016-1387-1 PMC4931696

[31] Thiele H, et al. Intraaortic balloon support for myocardial infarction with cardiogenic shock. N Engl J Med. 2012;367(14):1287–96. 22920912 10.1056/NEJMoa1208410

[32] Prondzinsky R, et al. Intra-aortic balloon counterpulsation in patients with acute myocardial infarction complicated by cardiogenic shock: the prospective, randomized IABP SHOCK trial for attenuation of multiorgan dysfunction syndrome. Crit Care Med. 2010;38(1):152–60. 19770739 10.1097/CCM.0b013e3181b78671

[33] Sheikh O, et al. Acute kidney injury in cardiogenic shock: a comprehensive review. Catheter Cardiovasc Interv. 2021;98(1):E91-e105. 32725874 10.1002/ccd.29141

[34] Wang D, et al. Admission proteinuria predicts the incidence of acute kidney injury among patients with acute ST-segment elevation myocardial infarction: a retrospective cohort study. Coron Artery Dis. 2024;35(3):215–20. 38436048 10.1097/MCA.0000000000001345

[35] Chen Y, et al. Dipstick proteinuria is a prognostic indicator of short-term mortality in patients with heart failure. Int J Cardiol. 2017;230:59–63. 28038813 10.1016/j.ijcard.2016.12.096

[36] Zilberberg MD, et al. Characteristics, hospital course, and outcomes of patients requiring prolonged acute versus short-term mechanical ventilation in the United States, 2014–2018. Crit Care Med. 2020;48(11):1587–94. 33045151 10.1097/CCM.0000000000004525

[37] Lai CC, et al. The outcomes and prognostic factors of patients requiring prolonged mechanical ventilation. Sci Rep. 2016;6:28034. 27296248 10.1038/srep28034 PMC4906399

[38] Marenzi G, et al. Acute kidney injury in ST-segment elevation acute myocardial infarction complicated by cardiogenic shock at admission. Crit Care Med. 2010;38(2):438–44. 19789449 10.1097/CCM.0b013e3181b9eb3b

[39] Lauridsen MD, et al. Trends in first-time hospitalization, management, and short-term mortality in acute myocardial infarction-related cardiogenic shock from 2005 to 2017: a nationwide cohort study. Am Heart J. 2020;229:127–37. 32861678 10.1016/j.ahj.2020.08.012

[40] Cherbi M, et al. Cardiogenic shock and chronic kidney disease: dangerous liaisons. Arch Cardiovasc Dis. 2024;117(4):255–65. 38594150 10.1016/j.acvd.2024.01.006

[41] Ostermann M, et al. Acute kidney injury. Lancet. 2025;405(10474):241–56. 39826969 10.1016/S0140-6736(24)02385-7

[42] Legrand M, Rossignol P. Cardiovascular consequences of acute kidney injury. N Engl J Med. 2020;382(23):2238–47. 32492305 10.1056/NEJMra1916393

[43] Meersch M, Zarbock A. Renal protection in the 21st century. Curr Opin Crit Care. 2016;22(6):554–9. 27811558 10.1097/MCC.0000000000000352

[44] Ronco C, et al. Cardiorenal syndrome. J Am Coll Cardiol. 2008;52(19):1527–39. 19007588 10.1016/j.jacc.2008.07.051

[45] Pöss J, et al. Risk stratification for patients in cardiogenic shock after acute myocardial infarction. J Am Coll Cardiol. 2017;69(15):1913–20. 28408020 10.1016/j.jacc.2017.02.027

[46] Wang JI, et al. Outcomes of hospitalizations for cardiogenic shock at left ventricular assist device versus non-left ventricular assist device centers. J Am Heart Assoc. 2020;9(23):e017326. 33222608 10.1161/JAHA.120.017326 PMC7763759

[47] Odutayo A, et al. AKI and long-term risk for cardiovascular events and mortality. J Am Soc Nephrol. 2017;28(1):377–87. 27297949 10.1681/ASN.2016010105 PMC5198285

[48] Joannidis M, et al. Lung-kidney interactions in critically ill patients: consensus report of the Acute Disease Quality Initiative (ADQI) 21 workgroup. Intensive Care Med. 2020;46(4):654–72. 31820034 10.1007/s00134-019-05869-7 PMC7103017

[49] De Oliveira-Gomes D, et al. Renal dysfunction across the spectrum of cardiogenic shock: mechanisms, clinical implications, and therapeutic strategies. Curr Heart Fail Rep. 2025;22(1):19. 40560292 10.1007/s11897-025-00706-z

